# Injectable *in situ* cross-linking chitosan-hyaluronic acid based hydrogels for abdominal tissue regeneration

**DOI:** 10.1038/s41598-017-02962-z

**Published:** 2017-06-02

**Authors:** Youming Deng, Jianan Ren, Guopu Chen, Guanwei Li, Xiuwen Wu, Gefei Wang, Guosheng Gu, Jieshou Li

**Affiliations:** Department of General Surgery, Jinling Hospital, Medical School of Nanjing University, Nanjing, P. R. China

## Abstract

Abdominal wall defect caused by open abdomen (OA) or abdominal trauma is a serious issue since it induces several clinical problems. Although a variety of prosthetic materials are commonly employed, complications occur including host soft tissue response, fistula formation and chronic patient discomfort. Recently, abundant natural polymers have been used for injectable hydrogel synthesis in tissue regeneration. In this study, we produced the chitosan - hyaluronic acid (CS/HA) hydrogel and investigated its effects on abdominal tissue regeneration. The physical and biological properties of the hydrogel were demonstrated to be suitable for application in abdominal wounds. In a rat model simulating open abdomen and large abdominal wall defect, rapid cellular response, sufficient ECM deposition and marked neovascularization were found after the application of the hydrogel, compared to the control group and fibrin gel group. Further, the possible mechanism of these findings was studied. Cytokines involved in angiogenesis and cellular response were increased and the skew toward M2 macrophages credited with the functions of anti-inflammation and tissue repair was showed in CS/HA hydrogel group. These findings suggested that CS/HA hydrogel could prevent the complications and was promising for abdominal tissue regeneration.

## Introduction

The abdominal cavity is commonly left open in a variety of situations potentially beneficial to patients with abdominal compartment syndrome (ACS) caused by infection or trauma^1–3^. In these settings, sometimes, abdominal cavity cannot be closed due to fascia retraction, which leads to relative abdominal wall defect. Furthermore, severe abdominal injuries can cause abdominal tissue loss, thereby forming absolute defect. Once the abdominal wall defect is formed, the abdominal viscera will be exposed to air, which may lead to visceral injury, infection and enteroatmospheric fistula. Reconstruction of the abdominal wall is a solution to these problems, thus, acceleration of abdominal tissue regeneration becomes the key process.

Prosthetic materials have been widely employed in hernia repair and temporary abdominal closure after open abdomen^4, 5^. Synthetic non-biodegradable biomaterials such as polypropylene (PP) and polyethylene meshes are commonly used due to their excellent mechanical properties. However, because of their poor biocompatibility, placement of these materials alone may induce host soft tissue response, fistula formation and chronic patient discomfort^6–8^.

Tissue engineering is designed to employ a biological degradable material or scaffold that enables tissue ingrowth and remodeling to result in wound repair and tissue regeneration. A variety of injectable hydrogels have been utilized as delivery systems, cell carriers, and scaffolds for tissue engineering^9–11^. Injectable hydrogels have the advantages of excellent malleability and filling property, therefore suitable for the irregular wound. Methods employed for the preparation of injectable *in situ* forming hydrogels vary from photopolymerization to chemical crosslinking^12, 13^. However, prolonged irradiation and the toxicity of the chemical crosslinking agents largely limit their applications. Recently, several polysaccharides such as dextran, chondroitin sulfate and hyaluronic acid (HA) were partially oxidized and then reacted with amino to form injectable *in situ* hydrogels for possible medical applications such as cartilage tissue engineering^14^ and adhesion prevention^15^.

HA is a liner glycosaminoglycan composed of repeating disaccharide units of N-acetyl-D glucosamine and D-glucuronic acid. HA widely exists in human body and is the major component of the extracellular matrix (ECM). Due to its good biocompatibility and biodegradability, HA shows excellent potential for application in drug delivery, wound healing and tissue engineering^13, 16–18^ but the rapid degradation of HA compromise its effectiveness for tissue engineering. Chitosan (CS) is a partially deacetylate derivative from chitin, and composed of glucosamine and N-acetyl-glucosamine. CS has been widely used in tissue engineering, wound healing, and other biomedical applications^19–22^ because of its biological properties such as biocompatibility, biodegradability, nontoxicity, and good cell-bound capability. However, CS has poor solubility in physiological solvents, which greatly limits its further application as an injectable scaffold.

Recently, CS and HA, after some modifications, have been employed to develop hybrid hydrogel for adhesion prevention and periodontal tissue engineering in several studies^15, 23, 24^. However, no research has examined the potential of CS/HA hydrogel to promote abdominal tissue regeneration. Our previous study has demonstrated that pre-formed hydrogel has the potential in acceleration of granulation tissue formation in open abdomen wounds^25^. Here, we produced an injectable CS/HA hydrogel and aimed to evaluate its effects on abdominal tissue regeneration. We studied its chemical synthesis, micromorphology, equilibrium swelling and degradation *in vitro*. Fibroblast cells were encapsulated within the hydrogels *in vitro* to assess cytocompatibility and potential applicability as cell carriers. Furthermore, the effectiveness in promoting the abdominal tissue growth in a rat model and its possible mechanisms were investigated in detail. For control purposes, commercial fibrin gel was similarly evaluated.

## Results

### Structure of polysaccharide derivatives

**Figure 1:**
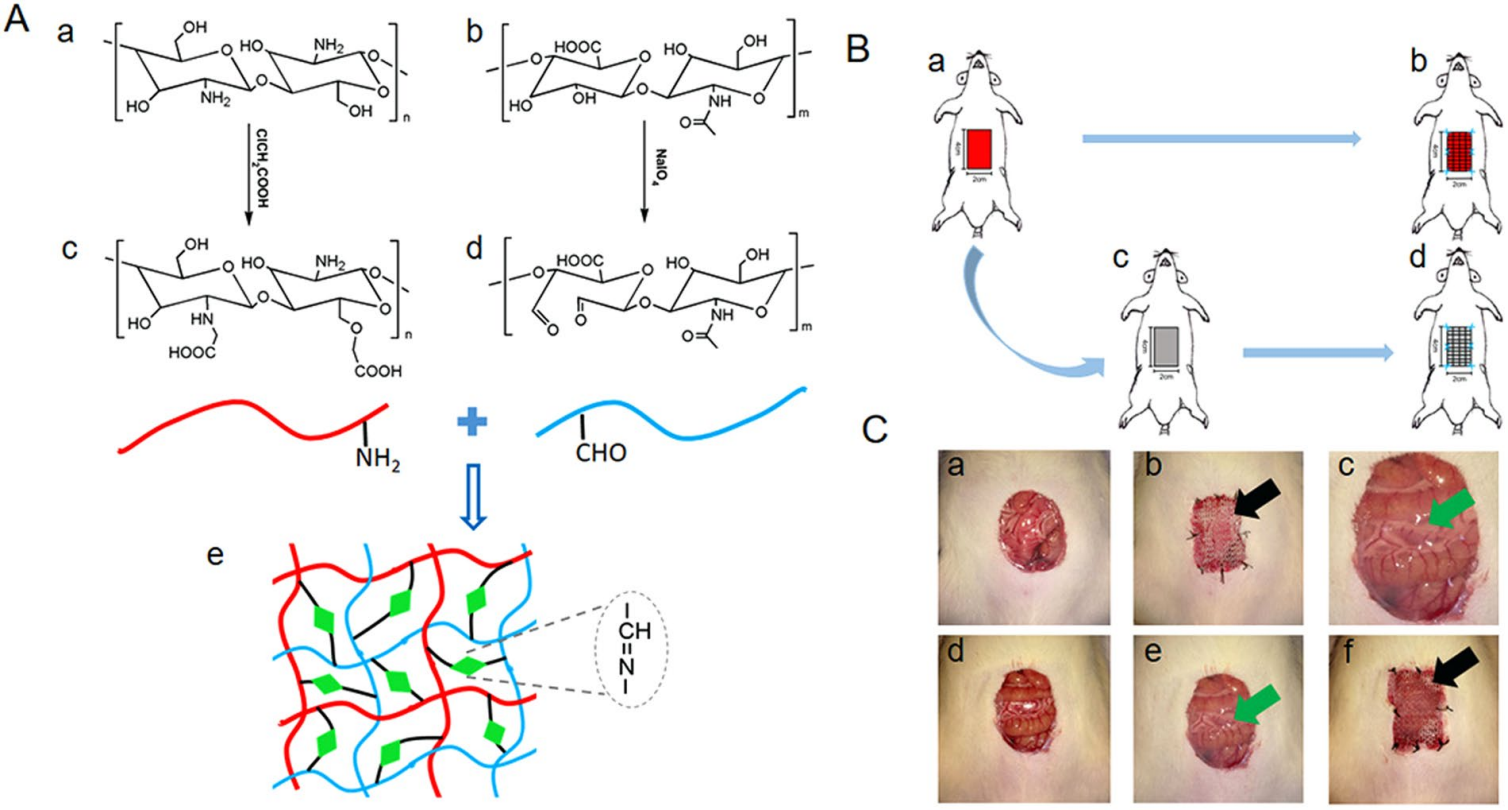
(**A**) Schematic illustration of preparation of CS/HA hydrogel via Schiff’s base reaction. Chemical structures of chitosan (CS) (a), carboxymethyl chitosan (NOCC) (c), hyaluronic acid (HA) (b), and aldehyde hyaluronic acid (A-HA) (d). (**B**) Schematic diagram of the animal model and the experimental procedure: (a) the abdominal wall defect model, (b) the Control group, (c) fibrin gel or CS/HA hydrogel employed over the defect, (d) the Fibrin gel or CS/HA hydrogel group. (**C**) Gross observation: (a,d) the established defect wound, (b) the Control group (black arrow: PP mesh), (c,e) the employed fibrin gel or CS/HA hydrogel (green arrow: fibrin gel or CS/HA hydrogel), (f) the Fibrin gel or CS/HA hydrogel group.

N, O-carboxymethyl chitosan (NOCC) was synthesized by carboxymethylation of CS. The carboxymethyl groups were introduced into the N-terminal and O-terminal of chitosan. One of the reactions is between the amine group of chitosan and electrophilic carbon atom of monochloroacetic acid. The other is between the hydroxyl group of chitosan and electrophilic carbon atom of monochloroacetic acid. The chemical structures of CS and NOCC are shown in Fig. 1A. The determined substitution degree of NOCC was 95%, which showed good water solubility in PBS (7.4). Aldehyde hyaluronic acid (A-HA) was obtained by oxidation reaction of HA using NaIO_4_. The vicinal hydroxyl groups of HA were oxidized to dialdehydes, thereby opening the sugar ring to form dialdehyde derivatives. The actual aldehyde content of A-HA was determined by hydroxylamine hydrochloride titration method, which revealed an extent of oxidation of 48.9%. The mechanism of gelation is via Schiff base reaction between amino of NOCC and aldehyde groups of A-HA. Formation of amide bond cross-linked NOCC with A-HA results in the formation of porous structure in the hydrogel.

FTIR spectra of CS, NOCC, HA and A-HA were displayed in Fig. S1A. Compared with the spectrum of CS, the spectrum of NOCC showed a characteristic peak (1608 cm^−1^) for the associated carboxylic acid salt (-COO^−^ asymmetric stretch) and a characteristic peak (1421 cm^−1^) that represented the symmetric stretching of the carboxylate C=O, suggesting that there were carboxymethyl groups existing on NOCC. Moreover, the spectrum showed that the peak intensity ratios (1029 cm^−1^/1057 cm^−1^) of the carboxymethylation of NOCC were decreased compared to that of CS, which was an indication that carboxymethyl groups were substituted to -CH2-OH at the C6 position of NOCC. The spectra of HA and A-HA were very similar and there was no signal detected corresponding to the aldehyde functionalities, which may be due to the formation of hemiacetals. The characteristic peak at 886 cm^−1^ was the result of hemiacetal structure in the spectrum of the CS/HA hydrogel.

### Gelation time of hydrogels

The CS/HA hydrogel was prepared by cross-linking NOCC with A-HA. The gelation time of different molar ratios of CS/HA hydrogels was determined by test tube inverting method, which were displayed in Table S1. As presented, the gelation time ranged from 70 s to 2400 s. The gelation time was largely decreased with the increase of A-HA. When the molar ratio was 1:2 (NOCC/A-HA), the gelation time was less than two minutes, which was suitable for injection on the abdominal wall defect and could completely cover the defects and adhere to the defects tightly. Therefore, this formulation was applied to perform the following studies.

Rheological properties of the CS/HA hydrogels were inspected in this study. Strain dependent oscillatory rheology (Fig. S1B) exhibited a broad linear viscoelastic region and a great anti-shear ability. Only when the strain was over 100%, the network of the hydrogel was broken, which indicated shear-thinning behavior. Frequency dependent oscillatory shear rheology (Fig. S1C) showed that G’ is dominant across the range of frequencies observed, indicating a hydrogel-like behavior. Time dependent oscillatory rheology displayed the G’ and G” as a function of time (Fig. S1D). As time went by, the G’ and G” increased, and G’ increased faster than G”. When G’ was higher than G”, the point (t_gel_ = 90 s) was determined as gelation time.

### Morphology of the CS/HA hydrogels

The micromorphology of the freeze-dried CS/HA hydrogel was characterized by SEM. The cross-sectional images of hydrogel was displayed in Fig. S2. It was clearly observed that the CS/HA hydrogel displayed a continuous and porous structure. This internal structure was potentially beneficial for nutrition infiltration and cellular growth.

### Equilibrium swelling and degradation of CS/HA hydrogels

Equilibrium swelling ratio of freeze-dried CS/HA hydrogels was determined gravimetrically in PBS (pH = 7.4) (Fig. S3A). As displayed, swelling ratio was increased rapidly in the first 2 hours and then the swelling rate slowed down. After 6-hour incubation, equilibrium swelling was achieved with a 34.8 fold increase to the weight of the dried hydrogels. Degradation of freeze-dried CS/HA hydrogels was determined as the incubation time in PBS (pH = 7.4) at 37 °C (Fig. S3B). During the incubation process, it was clearly observed that the hydrogel samples demonstrated a decrease in size. The initial degradation rate was slow in the first day due to the swelling effect. Subsequently, hydrogels underwent a faster degradation behavior. Over 60% weight loss of the hydrogels was observed at the 7th day.

### *In vitro* cytocompatibility

**Figure 2:**
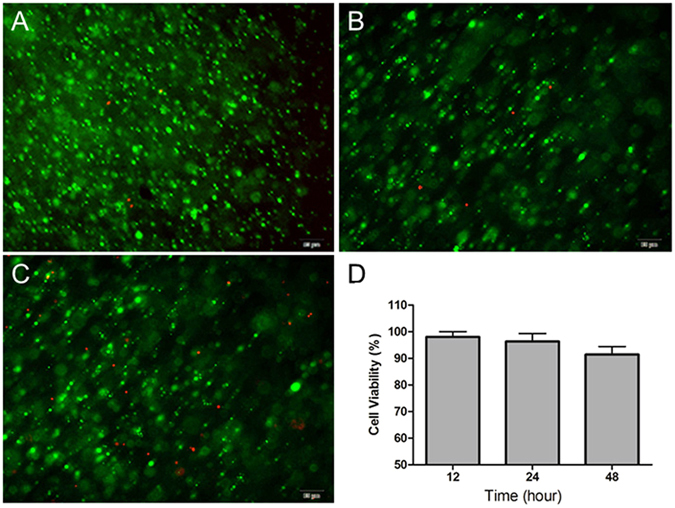
*In vitro* cytocompatibility of CS/HA hydrogel. Live/Dead staining of the encapsulated L929 fibroblasts for 12 h (**A**), 24 h (**B**), and 48 h (**C**). (**D**) The cell viability at different culture times.

Encapsulation of fibroblasts in CS/HA hydrogel was performed to explore the cytocompatibility and cytotoxicity since fibroblasts play a significantly important role in granulation tissue formation and tissue regeneration. From the Live/Dead staining of cells, a majority of fibroblasts encapsulated in the hydrogels were found to remain viable after 12 h, 24 h and 48 h culture (Fig. 2) and elliptical or round shaped fibroblasts were uniformly distributed in the hydrogels. After 48 h culture, dead cells were relatively increased, but the cell viability remained over 90%. The results suggested that CS/HA hydrogel demonstrated good cytocompatibility and non-toxicity. The interconnected porous structure of CS/HA hydrogel, which allowed nutrients and oxygen to infiltrate in, provided a suitable environment for fibroblasts.

### Histological analysis

**Figure 3:**
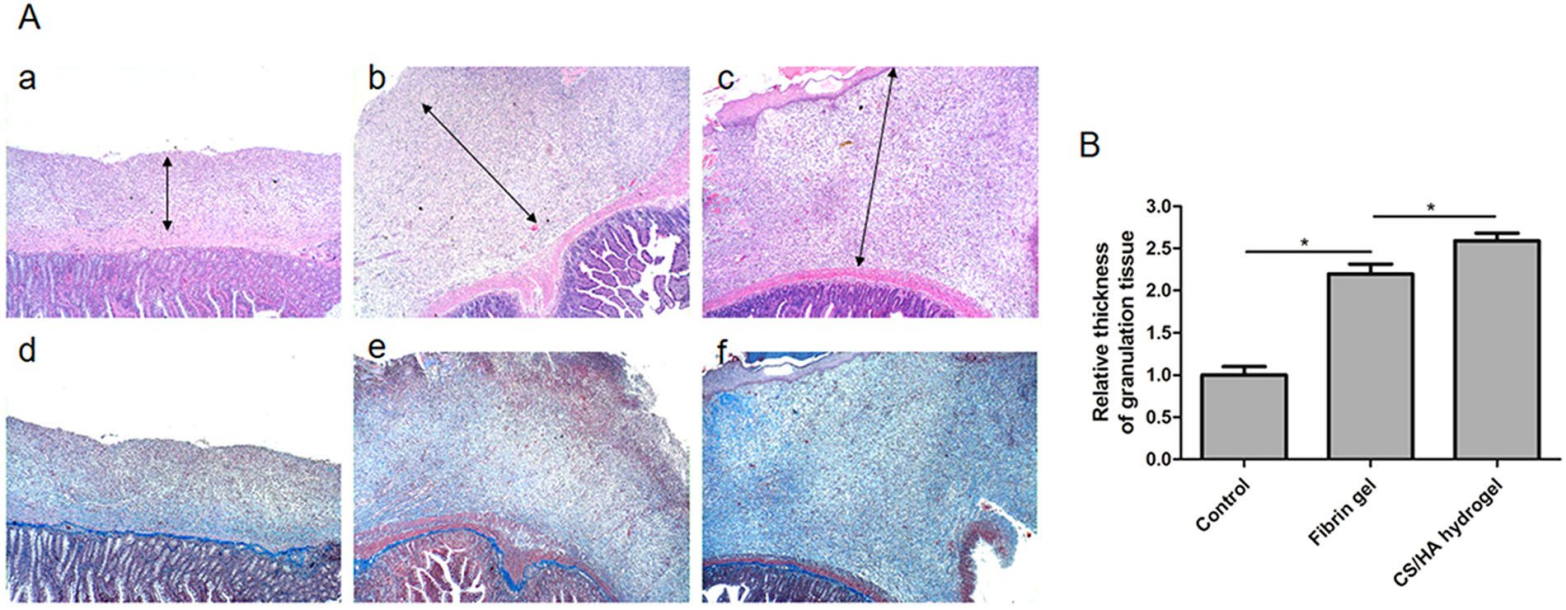
(**A**) Representative H&E and Masson’s trichrome stained histologic cross sections of different groups: the Control (a and d), Fibrin gel (b and e), and CS/HA hydrogel (c and f). Staining for the upper image is with H&E, and for the lower image with Masson’s trichrome. (**B**) The statistical analysis of the relative connective tissue thickness in the three groups. Data are presented as mean with standard deviation as error bars, *P < 0.05.

Abdominal regenerative tissue was completely formed by day 7, which was consistent with our previously study^25^. The regenerative tissues together with underlying bowel loops were stained with H&E and Masson trichrome (Fig. 3 and Fig. S4). In CS/HA hydrogel, faster tissue regeneration response was observed, which was characterized as much more cellular accumulation and matrix deposition. H&E staining depicted the connective tissue area and the underlying intestines. Relative thickness of the regenerative tissue was calculated. CS/HA hydrogel induced approximately 2.5 fold increase in thickness of the regenerative tissue and a great amount of fibroblasts and endothelial cells were observed. Inner tissue was depicted at high magnification. The capillaries were scattered in the regenerative tissue and compared to the Control and Fibrin gel, more capillaries were distributed in CS/HA hydrogel. Moreover, Masson’s trichrome staining showed overall distribution of collagen deposition in the regenerative tissue. The amount and density of collagen fibers were increased in CS/HA hydrogel.

### Neovascularization

**Figure 4:**
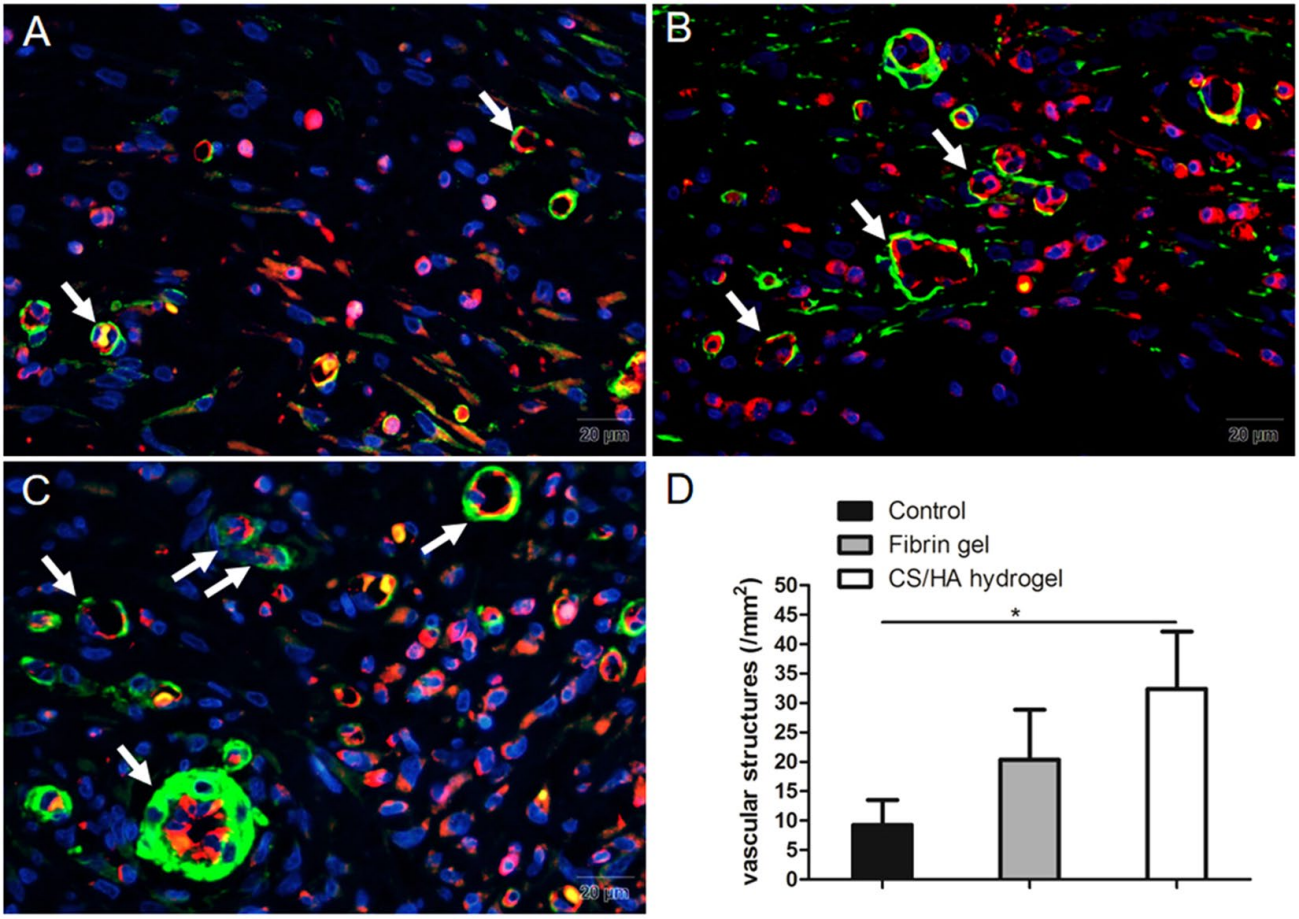
Neovascularization in different groups: (**A**) the Control; (**B**) Fibrin gel; (**C**) CS/HA hydrogel. The double staining revealed that CD31 (+) structures were surrounded by a-smooth muscle actin positive cells (indicated with white arrows). (**D**) Quantification of the CD31 labeled structures. Scale bar: 20 μm. *P < 0.05.

The vascular density in the regenerative tissue was assessed by CD31 immunostaining and α-SMA immunostaining was used to exhibit the presence of smooth muscle cells (Fig. 4 and Fig. S5). Few positive staining of CD31 was detected in the Control, whereas the Fibrin gel and CS/HA hydrogel demonstrated marked CD31 staining. These structures were remarkably distributed in CS/HA hydrogel. The double positive immunostaining showed that in many instances, the CD31 positive structures were surrounded by α-SMA positive cells. The spatial relationship between CD31 and α-SMA staining indicated mature vascular formation.

### Extracellular matrix deposition

**Figure 5:**
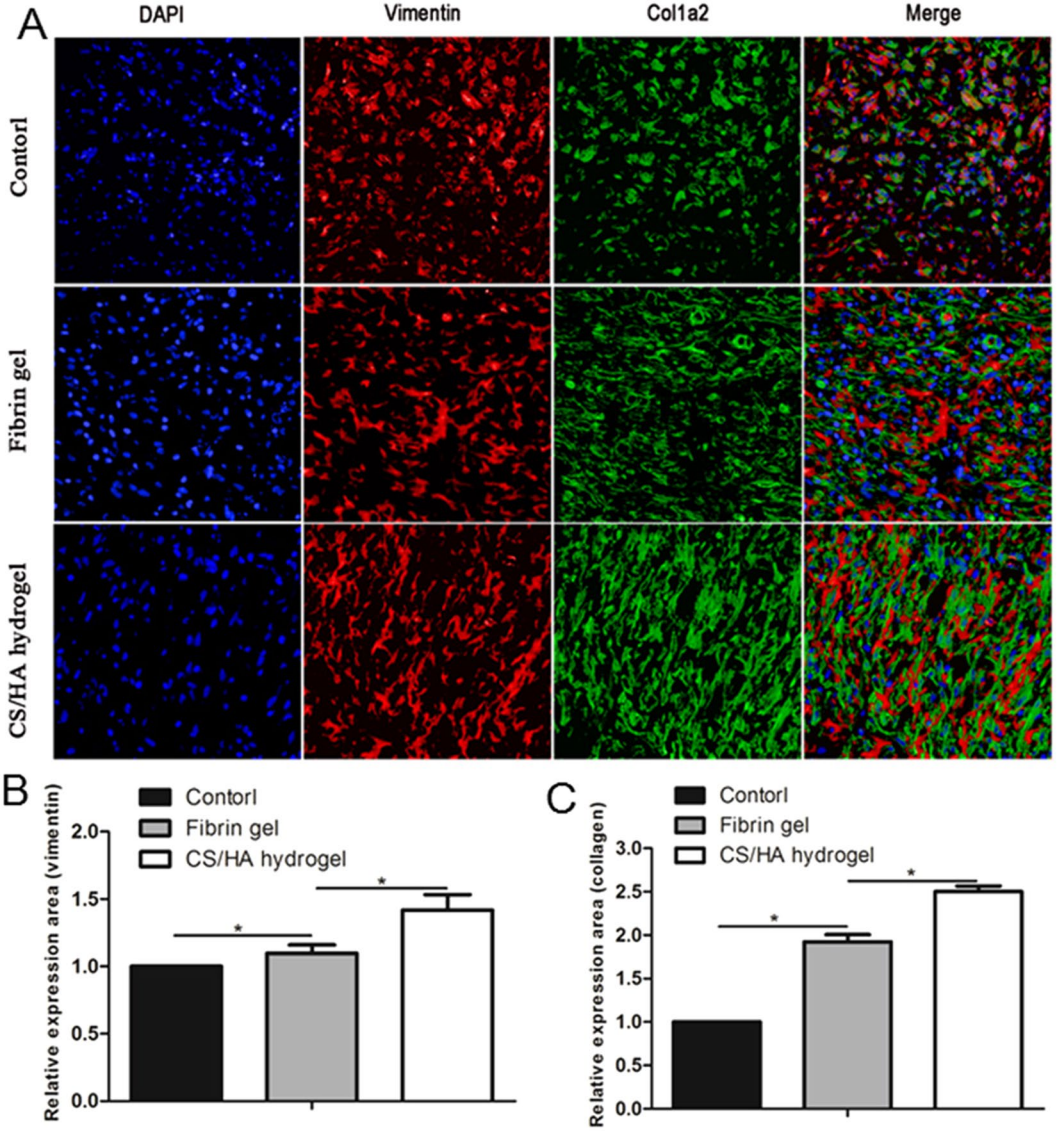
(**A**) Representative immunofluorescent images of collagen deposition in the three groups. DAPI (nuclei) = blue, Vimentin (fibroblasts) = red, Col1α2 (collagen) = green. The merge images revealed that collagen expression was found both in and around vimentin-expressing fibroblasts. (**B**) Quantification of vimentin expression. (**C**) Quantification of collagen expression.

Immunofluorescence staining of vimentin and col1a2 revealed the extracellular matrix (ECM) deposition during the formation of connective tissue (Fig. 5). Collagen was deposited substantially in Fibrin gel (1.92 ± 0.08 versus 1.00) and CS/HA hydrogel (2.50 ± 0.06 versus 1.00) while inapparently in the Control group, consistent with the findings in histological analysis. Double immunofluorescence staining showed that collagen expression was found both in and around vimentin-expressing fibroblasts. The spatial colocalization indicated that the main cellular sources of collagen expression were fibroblasts. Furthermore, for collagen angular distribution, compared with the random orientation of collagen fibers in Control, CS/HA hydrogel demonstrated a high degree of directional alignment of collagen.

### Macrophage phenotype

**Figure 6:**
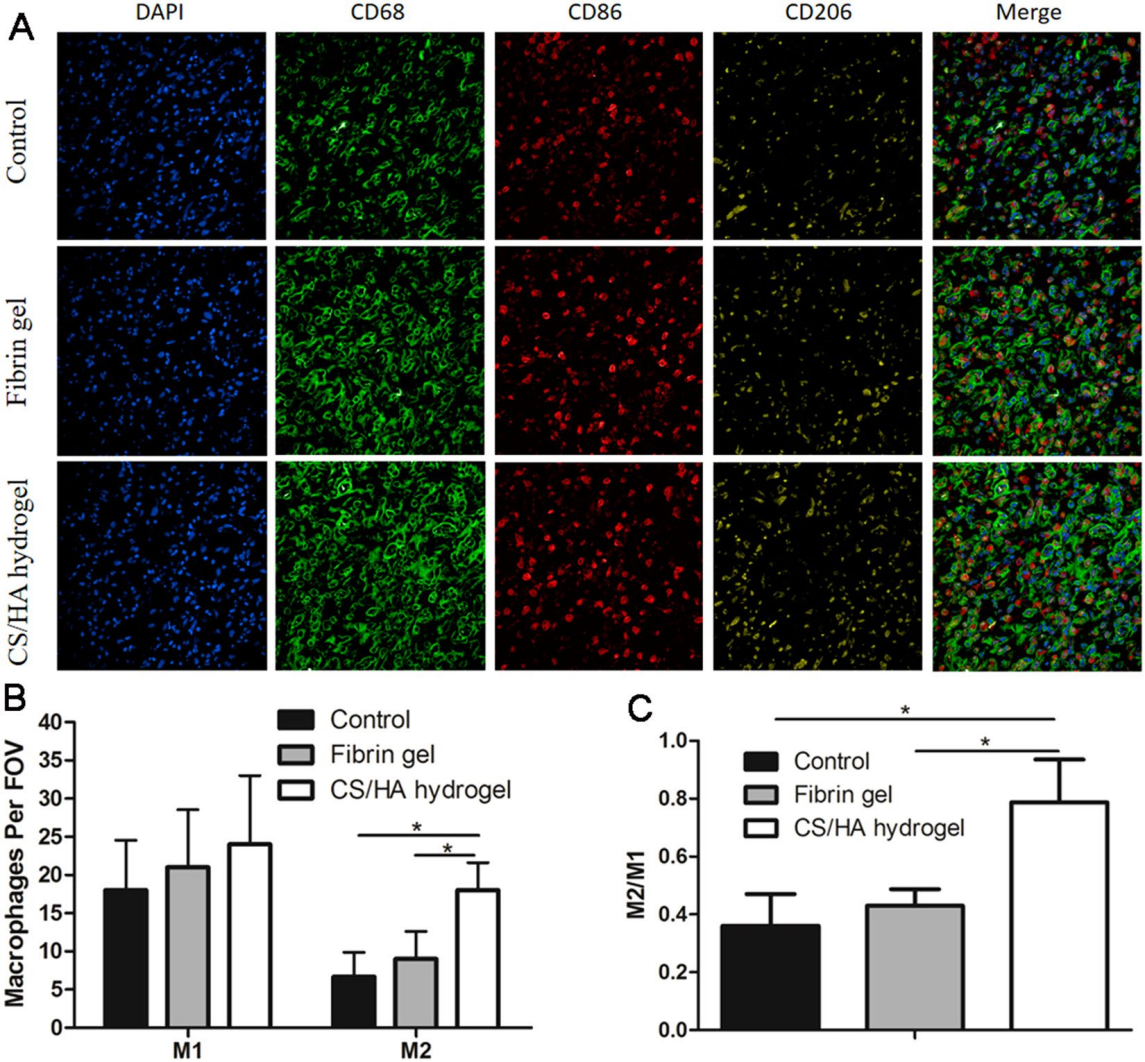
(**A**) Representative immunofluorescent images of the macrophages phenotype markers in three groups. DAPI (nuclei) = blue, CD68 (M0) = green, CD86 (M1) = red, CD206 (M2) = yellow. (**B**) Quantification of the pro-inflammatory M1 (CD86+/CD68+) macrophages and constructive remodeling M2 (CD206+/CD68+) macrophages. (**C**) Statistical analysis of the relative proportion of M2 and M1.

Immunofluorescence staining of pan-macrophages (CD68), pro-inflammatory M1 macrophages (CD86) and remodeling M2 macrophages (CD206) was used to investigate the effect of CS/HA hydrogel on macrophage polarization (Fig. 6A). The numbers of M1 pro-inflammatory cells were similar among the three groups (Fig. 6B). However, the CS/HA hydrogel markedly increased the number of M2 cells (Fig. 6B). Higher proportion of M2/M1 was an indication of skew toward to M2 cells (Fig. 6C).

**Figure 7:**
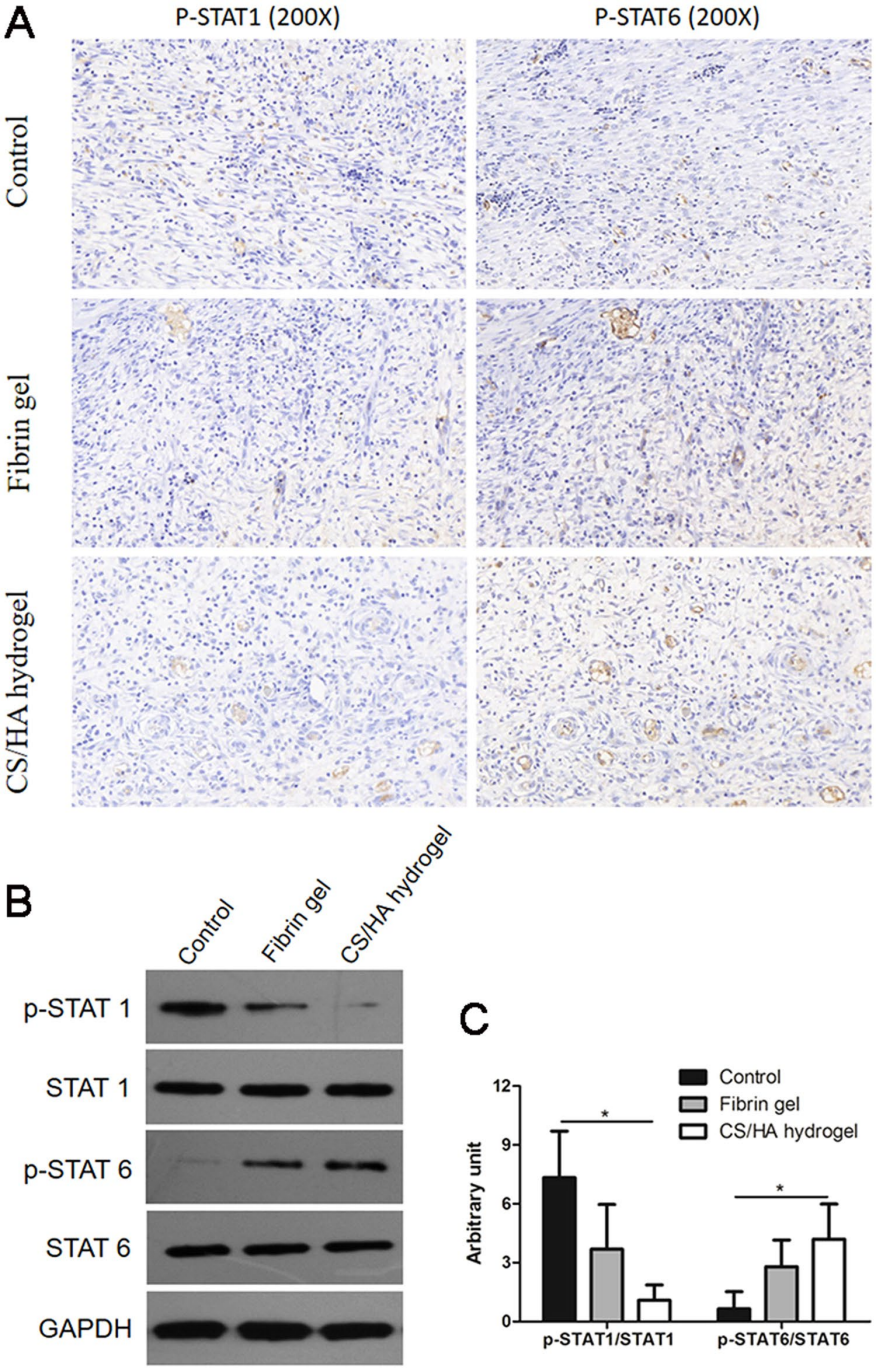
(**A**) Expression of p-STAT1 and p-STAT6 in the regenerative tissue was determined by immunohistochemistry. The amplification was 200× . (**B**) Representative western blot images (cropped) of expression of p-STAT1, STAT1, p-STAT6 and STAT6 in the regenerative tissue. (**C**) Gray intensity of p-STAT1 and p-STAT6 was analyzed. *P < 0.05.

To further investigate the possible mechanism of the macrophages polarization, the phosphorylation of STAT1 and STAT6 were measured (Fig. 7). Immunohistochemical assay revealed a decreased phosphorylation of STAT1 in the regenerative tissue of Fibrin gel and CS/HA hydrogel, compared to the Control. In contrast, phosphorylation of STAT6 was found relatively increased in Fibrin gel and CS/HA hydrogel (Fig. 7A). The results of western blot were consistent with the findings above (Fig. 7B and Fig. S6). CS/HA hydrogel showed significant increase of p-STAT6 and decrease of p-STAT1 (Fig. 7C).

### Gene expression

**Figure 8:**
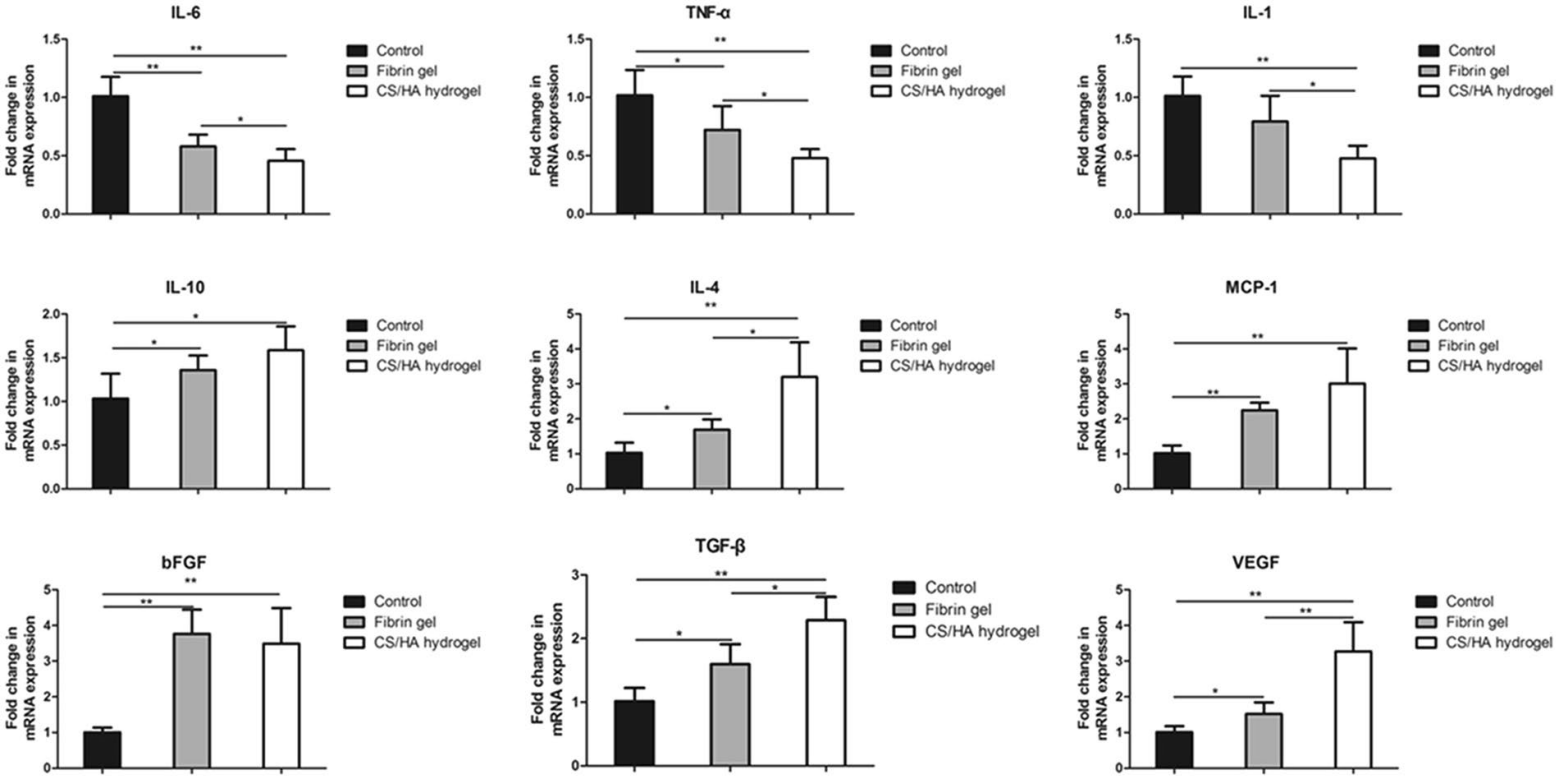
Quantitative analysis of gene expression in three groups. Relative expression of cytokines involved in tissue regeneration were expressed as fold change. Data are presented as mean with standard deviation as error bars, *P < 0.05, **P < 0.001.

Genes involved in the process of tissue formation were analyzed by qPCR (Fig. 8). Pro-inflammatory related factors interleukin (IL)-6 and tumor necrosis factor (TNF)-α increased significantly in the Control, whereas, anti-inflammatory factors IL-10 and IL-4 were markedly induced in Fibrin gel and CS/HA hydrogel. CS/HA hydrogel significantly upregulated the expression of IL-4 compared to both the Fibrin gel and the Control. Monocyte chemotactic protein (MCP)-1 expression were enhanced both in Fibrin gel and CS/HA hydrogel. For trophic and fibroplasia-related factors, basic fibroblast growth factor (bFGF) and transforming growth factor (TGF)-β were detected. TGF-β and bFGF were released markedly in Fibrin gel and CS/HA hydrogel compared to the Control. When compared to Fibrin gel, secretion of TGF-β was more remarkable in CS/HA hydrogel. In addition, angiogenesis related factor VEGF was significantly enhanced in CS/HA hydrogel.

## Discussion

OA caused by ACS and abdominal wall tissue loss due to traumatic injury tissue loss contribute to abdominal wall defects, where spontaneous tissue regeneration cannot be achieved and abdominal viscera are exposed to air. Various prosthetic materials were used on the abdominal wall defects that are required to have mechanical strength^26, 27^. However, de novo functional abdominal tissue regeneration is not achievable and due to direct contact with the intestines, they may cause bleeding, intestinal wall erosion and even fistula. Biological response materials may provide a solution to this problem through interaction with host cells to promote abdominal tissue regeneration. Our previous report had demonstrated that hydrogel as a coating to PP mesh could minimize the damage of PP mesh implantation on the intestines and accelerate the granulation tissue formation in open abdominal wounds^25^. These results provide a perspective that hydrogel has the potential to be used on the abdominal defects. In the previous study, we prepared a pre-formed composite material through coating chitosan/gelation hydrogel to PP mesh, but a long preparation time of the pre-formed composite material and improper adaptation to the irregular defects may hinder further application. Furthermore, what have not been clear are that how the hydrogel performs in the process of tissue regeneration and whether the hydrogel provides more benefit over the prosthetic materials alone and commercial fibrin gel? In this study, we prepared an injectable in situ hydrogel based on CS and HA to solve the above problems and to evaluate the effects on tissue regeneration. The possible mechanism was also discussed.

Injectable hydrogels have gained wide applications in adipose, bone and cartilage engineering for their easy handling and complete filling of defective area. The injectable fibrin gel has been employed in all areas of surgery and explored for the potential in cardiac tissue engineering due to its formation of fibrin meshwork and inherent binding domains for the growth factors^28, 29^. Considering the limited availability of autologous fibrin and the risk of infectious transmission of xenogenous fibrin, abundant natural polymers have been used for injectable hydrogel applications in tissue engineering. HA can provide the ECM-mimic microenvironment for the cells and promote angiogenesis^17, 30^. Furthermore, HA has several binding sites of CD44 and the interaction between CD44 of cells and HA was found to promote migration of mesenchymal stem cells^31^. Based on these, CS/HA hydrogel has gained the basic evidence for cells recruitment to defect site from the host.

In this study, an injectable in situ hydrogel was formed after Schiff’s base reaction between the modified CS and HA. Hydrogel of molar ratio 1:2 (CS: HA) was chosen for its proper gelation time, which was suitable for in situ injection in abdominal defect. The G’ of the optimized hydrogel (34 Pa) was lower than that reported by previous studies^15, 23^, which might be due to different source of CS and HA and preparation process. The CS/HA hydrogel was softer but it was robust for application in abdominal wall defect. The three-dimensional (3D) porous structure and swelling property of the hydrogel could facilitate nutrition infiltration and cellular ingrowth. *In vitro* degradation test showed that the rate of degradation matched with the rate of abdominal tissue infiltration, which could allow hydrogel to be degraded with regenerative tissue ingrowth. *In vitro* cytocompatibility evaluation displayed high viability of encapsulated fibroblasts, which indicated good biocompatibility and the potential as cell carriers.

A rat model of abdominal wall defect was established to evaluate the effects on abdominal tissue growth. One week after the surgery, the regenerative tissue was formed on the defect, which was consistent with previous studies^25, 32^. CS/HA hydrogel induced approximately 2.5 fold increase in thickness of regenerative tissue compared to the Control. Substantial cellular infiltration was responsible for tissue thickness and further remolding^33^. The increase in tissue thickness indicated faster cellular response and acceleration of tissue formation. Generally, in the process of tissue regeneration, host cells involved in angiogenesis and tissue repair are recruited to facilitate new tissue and vessel formation^34^. Abundant fibroblasts and endothelial cells observed in CS/HA hydrogel provided evidence for the faster tissue formation. Masson’s trichrome staining and double immunofluorescence staining of vimentin and col1a2 revealed that the amount of collagen fibers were significantly increased in CS/HA hydrogel and further, for collagen angular distribution, CS/HA hydrogel displayed high degree of directional alignment. Collagen is an important element of the natural extracellular matrix (ECM) and widely exists in mammalian tissues. Collagen deposition is one of the key process for tissue regeneration and restoration of structure and function^35^. Insufficient amounts of collagen deposition may lead to weak tissue strength. Sufficient deposition and directional alignment of collagen fibers in CS/HA hydrogel provided excellent anatomical structure, which was essential for lateral tissue remolding.

Sufficient blood supply is crucial in the tissue regeneration. Thus, hydrogels that aimed to mimic native tissue or ECM should enable angiogenesis. Delayed vascularization or lack of vascularization may hamper tissue regeneration^36^. In this study, remarkable vessels were found distributed in CS/HA hydrogel and almost all the vessels showed mature morphology. Given the high expression of VEGF in CS/HA hydrogel, it was reasonable to believe that CS/HA hydrogel promoted angiogenesis. HA-based hydrogels have been demonstrated to improve angiogenesis in the process of cardiac repair and support host cells for their reparative functions^37^. The 3D porous structure allowed nutrient supply and oxygen infiltration required by cells growing inside the hydrogel, which is the possible reason for the enhanced vascularization.

In the process of hydrogel-aided wound healing and tissue regeneration, some mechanisms are involved including the comparable rate of degradation of the hydrogel, recruitment of host cells to the site of hydrogel and the macrophages phenotype modulation. Macrophages play an important role in tissue repair and remodeling during wound healing. In particular, M2 macrophages are credited with the functions of anti-inflammation, tissue repair and remodeling^38^. Results from the present study found that in CS/HA hydrogel, the number of M2 cells significantly increased. The M2/M1 analysis revealed the skew toward M2 cells. Generally, M1 cells are producers of inflammatory cytokines (IL-6, TNF-α) while M2 cells expressed IL-10^39^. The high level of IL-10 and low levels of IL-6 and TNF-α in CS/HA hydrogel may be the consequence of macrophages phenotype modulation. Activated M2 cells play an important role in fibrogenesis. TGF-β production in macrophages can promote collagen deposition and in turn, TGF-β can stimulate skew of M2 macrophages^40^. In this study, TGF-β together with trophic factors bFGF and VEGF were significantly up-regulated in CS/HA hydrogel, which enhanced the recruitment of fibroblasts for tissue growth and production of extracellular matrix. To confirm skew of macrophages phenotype, phosphorylation of STAT1 and STAT6 were evaluated. A predominance of STAT1 activation promotes M1 macrophage polarization, in contrast, remarkable STAT6 activation results in M2 macrophage polarization^41^. Results from this work found CS/HA hydrogel improved the phosphorylation of STAT6 while phosphorylation of STAT1 was negligible. It suggested the regulation of activation of STAT1 and STAT6 may contribute to the skew of macrophage phenotype.

As a preliminary study, several limitations need to be addressed. First, the batch to batch variations in natural polymers CS and HA may exist due to their different origins, which might hinder further clinical translation of the CS/HA hydrogel. Second, this study just focused on the early process of tissue regeneration after application of the CS/HA hydrogel, and has not evaluated the subsequent effects on regenerative tissue function, skin graft and associated complications. In the next stage, consequences of the observed results need to be further examined.

## Conclusions

The injectable *in situ* forming CS/HA hydrogels were produced via Schiff’s base reaction and demonstrated to be suitable for the application in open abdomen and abdominal wall defect. In a rat model, CS/HA hydrogel promoted abdominal tissue formation via substantial cellular infiltration and sufficient ECM deposition. Neovascularization was predominantly enhanced in CS/HA hydrogel and the skew of M2 macrophages in CS/HA hydrogel was confirmed. Cytokines involved in angiogenesis and corresponding cellular response were improved in CS/HA hydrogel. These results indicate that the CS/HA hydrogel has potential in abdominal tissue regeneration.

## Materials and Methods

### Materials, cell lines, and animals

Chitosan (deacetylation degree: 85%, Mw: 2 × 10^5^), hyaluronic acid sodium (Mw: 2 × 10^5^), sodium periodate, monochloroacetic acid and ethylene glycol were purchased from Sigma–Aldrich (USA). Polypropylene mesh was purchased from Budd Company (Troy, MI, USA). Fibrin gel was purchased from Hangzhou universal medical technology development co., LTD (China). All other reagents were of analytical reagent grade.

L929 cells were purchased from Nanjing keygen technology development co., LTD (China) and cultured in Eagle’s Minimum Essential Medium (EMEM, Gibco, USA) with 10% fetal bovine serum (FBS, Gibco, USA) at 37 °C with a humidified 5% CO_2_ atmosphere.

Adult male Sprague-Dawley rats (200–250 g) were provided from Jinling Hospital (Nanjing, China). The animals were maintained at a temperature of 25 °C and a relative humidity of 50–60% under natural light–dark cycles and allowed free access to food and water. All the animal care and experimental protocols were reviewed and approved by Animal Investigation Ethics Committee of Jinling Hospital. All methods were performed in accordance with the relevant guidelines and regulations.

### Modifications of CS and HA

Synthesis of N, O-carboxymethyl chitosan (NOCC) was prepared as previously described with some modifications^42, 43^. Briefly, 5 g CS was suspended into 50 mL of isopropyl alcohol and the resulting slurry was stirred in a flask at room temperature. A 25 mL of aqueous NaOH solution (10 mol/L) was dropwisely added to the stirred slurry over a period of 15 min and the resulting mixture was stirred for additional 30 min. Subsequently, 50 mL of aqueous monochloroacetic acid (60% w/v) was dropwise added in five equal portions over a period of 10 min. The mixture was heated with stirring at 60 °C for 3 h. The reaction mixture was neutralized using HCl solution (10%), then, the mixture solution was filtered. The residue solid product (NOCC) was thoroughly rinsed three times with methanol and two times with alcohol. The dry product was obtained in a freeze drier.

Aldehyde hyaluronic acid (A-HA) was synthesized according to an already reported procedure slightly modified^44, 45^. Briefly, 1 g HA (2.5 mmol) was dissolved in 100 mL double distilled water at a concentration of 10 mg/ml. An aqueous solution of sodium periodate (2.5 mmol, 5 mL) was added dropwise, and the reaction was stirred for 2 h at room temperature in the dark. 1 ml ethylene glycol was added to quench the unreacted sodium periodate. The reaction was stirred for another 1 h at room temperature and the solution was purified by exhaustive dialysis (MWCO 12,000) against H_2_O for 3 days, three times per day and the dry product was obtained by freeze-drying.

The percentage oxidation of A-HA was determined by measuring the aldehyde content using hydroxylamine hydrochloride titration method as previously described^46^.

### Characterization of NOCC and A-HA

Fourier transform infrared spectra (FTIR) was used to confirm the substitutions of carboxymethyl groups on the amino and hydroxyl sites of NOCC and aldehyde groups in A-HA. FTIR spectra of CS, NOCC, HA and A-HA were evaluated. FT-IR spectra were performed using a Nicolet-6700 spectrometer from Thermo Electron at room temperature, in the wave number range of 4000–500 cm^−1^ by KBr pellet technique. The powders were ground to dry KBr disk and 32 scans at a resolution of 4 cm^−1^ were used to record the spectra.

### Preparation and characterization of CS/HA hydrogels

#### Preparation

CS/HA hydrogels were prepared by cross-linking NOCC with A-HA. Briefly, NOCC and A-HA were dissolved in normal saline and the total concentration of NOCC and A-HA was fixed as 4.5% Wt. The cross-linked composite hydrogel were prepared by mixing NOCC and A-HA solution at molar ratio of 1/2, 1/1, 2/1 at room temperature.

#### Infrared (IR) spectroscopic measurement

FTIR was applied to assess the synthesis of the CS/HA hydrogel. FTIR spectra was recorded with Nicolet-6700 spectrometer from Thermo Electron against a blank KBr pellet background at room temperature.

#### Rheological properties

Rheological properties of CS/HA hydrogel was measured by an Anton Paar MCR301 rheometer equipped with a 25 mm plate−plate. For these studies, storage modulus (elastic modulus, G’) and loss modulus (viscous modulus, G”) were both analyzed. The constant frequency was fixed at 10 Hz at the oscillatory strain sweep experiments. The constant strain was fixed at 1% for oscillatory frequency sweep experiments. For oscillatory time sweep experiments, the constant strain and frequency were fixed at 1% and 10 Hz, respectively. The gelation time was determined as the time when G’ became higher than G”.

#### Morphology

The morphology of CS/HA hydrogel was characterized by scanning electron microscopy (SEM) after gelation. Then the hydrogel was freeze-dried and cryo-fractured in liquid nitrogen. After coated with a thin layer of gold on cross-sectional surface, the surface and cross-sectional morphologies were viewed using an S-4800 SEM (Hitachi Ltd., Japan) operated at 5.0 KV accelerating voltage.

#### Equilibrium swelling

Swelling ratio of the freeze-dried hydrogel was measured gravimetrically. The known weights of freeze-dried hydrogels formed from A-HA (60 mg/ml, w/v) and NOCC (30 mg/ml, w/v) were immersed in phosphate buffered saline (PBS, pH = 7.4). At predetermined time point, the swollen hydrogels were removed and weighed after the excess of water lying on the surface was absorbed with a filter paper. The equilibrium of swelling was achieved when the weights of samples kept constant. All experiments were done in triplicate. The equilibrium swelling ratio (ESR) was calculated using the following equation:

$${\rm{ESR}}=({{\rm{W}}}_{{\rm{t}}}\,-\,{{\rm{W}}}_{{\rm{d}}})/{{\rm{W}}}_{{\rm{d}}}$$

where W_t_ and W_d_ were the weights of hydrogels at the equilibrium swelling state and at the dry state.

#### In vitro degradation

The *in vitro* degradation of CS/HA hydrogels was examined gravimetrically under simulated physiological conditions. Briefly, 1 ml of the hydrogel formed at a molar ratio of 1/2 (NOCC/ A-HA) was lyophilized and weighed (W_0_). Weight loss of W_0_ was monitored as a function of incubation time in PBS (pH = 7.4) at 37 °C. At specific time point, the samples were carefully withdrawn from the PBS. Then, the samples were freeze-dried and weighed (W_t_). The weight loss percentage (ΔW %) was defined as following equation:

$${\rm{\Delta }}{\rm{W}}( \% )=({{\rm{W}}}_{{\rm{0}}}{-{\rm{W}}}_{{\rm{t}}})/{{\rm{W}}}_{{\rm{0}}}\times \mathrm{100} \% $$

### *In vitro* cytocompatibility analysis

To evaluate the cytocompatibility of CS/HA hydrogel, L929 fibroblasts were encapsulated in the injectable hydrogels under sterile conditions. Briefly, NOCC and A-HA solutions at the molar ratio of 1/2 in PBS were sterilized by filtration through filters with a pore size of 0.22 mm. To encapsulate fibroblasts, 0.15 ml of A-HA solution was mixed with fibroblast/medium suspension (0.15 ml), then, 0.3 ml of NOCC solution was added. The cell density in the gel was fixed at 1.0 × 10^6^ cells/ml. The mixture solution was immediately injected into a 24-well plate before the formation of hydrogels and incubated at 37 °C for 30 min to form a composite cells/hydrogel matrix. 0.5 ml EMEM medium containing 10% FBS was added and the samples were cultured at 37 °C with a humidified atmosphere of 5% CO_2._ All experiments were done in triplicate.

Cell survival in the hydrogels were studied using a live–dead assay. At 12 h, 24 h and 48 h, the cell/hydrogel matrix were stained with Calcien AM and propidium iodide according to the manufacturers’ instructions. The viability of cells inside the hydrogels was observed under a Leica fluorescence microscope. As a result, live cells fluoresce green and dead cells fluoresce red.

### Surgical model and experimental protocol

An abdominal wall defect model was established as previously described with slight modification^25^. Briefly, after fasted overnight, all rats were anesthetized through intraperitoneal injections of a ketamine (50 mg/kg body weight) and xylazine (5 mg/kg body weight) mixture. Then the middle abdomen was prepared for aseptic surgery by clipping the fur over the entire abdominal region, and cleaning the operative area with medical iodine volts for 3 times and 75% alcohol for two times, followed by placing sterile surgical drapes over the entire field. A 2 × 4 cm partial abdominal defect was created by removing full thickness abdominal wall. After that, the animals were randomly divided into three groups: Control group, Fibrin gel group and CS/HA hydrogel group with eight rats in each group. For Fibrin gel group and CS/HA group, 1 ml of fibrin gel and CS/HA (1/2) hydrogel were injected to cover the defects, respectively. Then, PP mesh (2 × 4 cm) were fixated directly on the edges of the defect using eight interrupted sutures (4/0) to achieve temporary closure for both the three groups. The experimental process is schematically illustrated in Fig. 1. The animals were put back to cages and allowed free access to food and water.

Seven days post-surgery, animals were sacrificed and abdominal granulation tissues on defect were excised with underlying bowel loops. Each sample of granulation tissue was divided into three parts. The part with underlying bowel loops was immersed in 10% neutral formaldehyde for further histology and immunohistochemistry analysis. The other two parts were carefully removed the bowel loops. Then, one part was for immunofluorescent staining and another part was for Quantitative Polymerase Chain Reaction (qPCR) and Western Blot (WB).

### Effectiveness of CS/HA hydrogel on abdominal tissue regeneration

#### Histology and immunohistochemistry

The granulation tissues together with underlying bowel loops used for histology were fixed with 10% neutral formaldehyde. The samples were removed from the formaldehyde, followed by dehydration and then embedded in paraffin. Serially section was performed using a microtome according to standard protocols. The sections, 5 μm in thickness, were processed for hematoxylin and eosin staining (H&E), Masson’s trichrome staining and immunohistochemical evaluation. Sections for immunohistochemistry were stained with p-STAT1 (Santa Cruz, sc-135648) and p-STAT6 (Santa Cruz, sc-11762). For neovascularization evaluation, sections were reacted with primary antibodies CD31 (KEYGEN, KGYM0118–7) and α-smooth muscle actin (α-SMA) (KEYGEN, KGYT5053-6) overnight at 4 °C. Sections were washed and incubated with TRITC- (KEYGEN, KGAA98) and FITC- (KEYGEN, KGAA26) conjugated secondary antibodies. Sections were rinsed and mounted with 4, 6-diamidino-2-phenylindole (DAPI) mounting medium to label Nuclei.

#### Immunofluorescence staining

The frozen samples were embedded in optimum cutting temperature solution and fixed in acetone, then, incubated with blocking buffer. Sections were incubated with primary antibodies col1a2 (Abcam, ab96723) and vimentin (Abcam, 200 ab20346) for 2 hours at room temperature, and then incubated with Alexa Fluor 488- and 555-conjugated secondary antibodies for 1 hour. Then, slides were rinsed and mounted with DAPI mounting solution. The collagen positive area and vimentin positive area were analyzed by Image-Pro plus 6.0 (Media Cybernetics, America), then, the positive areas relative to cell numbers were calculated respectively. Three field of view were selected and results were expressed by relative magnitude. To characterize macrophage phenotype, Sections were incubated with primary antibodies overnight at 4 °C. Primary antibodies against the pan-macrophage marker CD68 (Abcam, ab955), the M1 macrophage marker CD86 (Abcam, ab53004), and the M2 macrophage marker CD 206 (Santa Cruz, sc-34577) were used. Sections were washed and incubated with Alexa Fluor 488-, 555- and 647- fluorescently conjugated secondary antibodies. Nuclei were labeled with DAPI. Photographs were acquired and analyzed with an Opera Phenix (PerkinElmer Inc., UK).

#### Quantitative polymerase chain reaction

The expression of corresponding genes related to process of granulation tissue formation were analyzed by qPCR. Primer sequences of detected genes were displayed in Table S2. The analysis was performed as previously described^31^. Briefly, Total RNA was isolated from tissues using TRIzol reagent (Invitrogen 15596-026, America). RNA quantity and purity were determined with a spectrophotometer (SHIMADZU UV-2450, Japan). cDNA synthesis was performed with PrimeScript RT reagent kit (Thermo Fisher K1622, America) according to manufacturer’s instructions. GAPDH was selected as the reference gene. The real-time PCR reaction was carried out for detecting genes expression using Real time PCR Master Mix (SYBR Green) (TOYOBO QPR-201, Japan) according to manufacturer’s instructions. C_t_ values were normalized to the reference gene to obtain the relative gene expression. Results were expressed by relative fold difference.

#### Western blot

Protein lysates obtained from the lysed tissue homogenate were separated by 10% SDS-PAGE and electrophoretically transferred onto Nitrocellulose (NC) membranes. The membranes were blocked with 5% nonfat milk for 1.5 h at room temperature, followed by incubation with primary antibodies overnight at 4 °C and then incubated with secondary antibody. Protein bands were visualized using a G: BOX Chemi XR5 Gel imaging and the gray intensity was analyzed with Gel-pro 32 analysis software.

### Statistical analysis

All statistical analysis was performed using IBM SPSS Statistics 19. Results were expressed as mean ± SD. One-way ANOVA analysis and Student’s t test were used to evaluate the differences between various experimental and control groups. P value of <0.05 was considered statistically significant.

## Electronic supplementary material

supplementary information

## References

[1] Carlson GL (2013). Management of the open abdomen: a national study of clinical outcome and safety of negative pressure wound therapy. Annals of surgery.

[2] Carr JA (2013). Abdominal compartment syndrome: a decade of progress. Journal of the American College of Surgeons.

[3] Schecter WP, Ivatury RR, Rotondo MF, Hirshberg A (2006). Open abdomen after trauma and abdominal sepsis: a strategy for management. Journal of the American College of Surgeons.

[4] Campanelli G, Catena F, Ansaloni L (2008). Prosthetic abdominal wall hernia repair in emergency surgery: from polypropylene to biological meshes. World journal of emergency surgery: WJES.

[5] Huang Q, Li J, Lau WY (2016). Techniques for Abdominal Wall Closure after Damage Control Laparotomy: From Temporary Abdominal Closure to Early/Delayed Fascial Closure-A Review. Gastroenterology research and practice.

[6] Sriussadaporn S, Sriussadaporn S, Kritayakirana K, Pak-art R (2006). Operative management of small bowel fistulae associated with open abdomen. Asian journal of surgery/Asian Surgical Association.

[7] Bellows CF, Alder A, Helton WS (2006). Abdominal wall reconstruction using biological tissue grafts: present status and future opportunities. Expert review of medical devices.

[8] O’Dwyer PJ (2005). Randomized clinical trial assessing impact of a lightweight or heavyweight mesh on chronic pain after inguinal hernia repair. The British journal of surgery.

[9] Phan VH, Thambi T, Duong HT, Lee DS (2016). Poly(amino carbonate urethane)-based biodegradable, temperature and pH-sensitive injectable hydrogels for sustained human growth hormone delivery. Scientific reports.

[10] Komeri, R. & Muthu, J. In situ crosslinkable elastomeric hydrogel for long-term cell encapsulation for cardiac applications. *Journal of biomedical materials research. Part A*. doi:10.1002/jbm.a.35833 (2016). 10.1002/jbm.a.35833 27409990

[11] Thorpe AA, Creasey S, Sammon C, Le Maitre CL (2016). Hydroxyapatite nanoparticle injectable hydrogel scaffold to support osteogenic differentiation of human mesenchymal stem cells. European cells & materials.

[12] Hardy JG, Lin P, Schmidt CE (2015). Biodegradable hydrogels composed of oxime crosslinked poly(ethylene glycol), hyaluronic acid and collagen: a tunable platform for soft tissue engineering. Journal of biomaterials science. Polymer edition.

[13] Leach JB, Bivens KA, Collins CN, Schmidt CE (2004). Development of photocrosslinkable hyaluronic acid-polyethylene glycol-peptide composite hydrogels for soft tissue engineering. Journal of biomedical materials research. Part A.

[14] Tan H, Chu CR, Payne KA, Marra KG (2009). Injectable *in situ* forming biodegradable chitosan-hyaluronic acid based hydrogels for cartilage tissue engineering. Biomaterials.

[15] Li L (2014). Biodegradable and injectable *in situ* cross-linking chitosan-hyaluronic acid based hydrogels for postoperative adhesion prevention. Biomaterials.

[16] Zhang Y, Heher P, Hilborn J, Redl H, Ossipov DA (2016). Hyaluronic acid-fibrin interpenetrating double network hydrogel prepared in situ by orthogonal disulfide cross-linking reaction for biomedical applications. Acta biomaterialia.

[17] Park HJ (2016). Catechol-Functionalized Hyaluronic Acid Hydrogels Enhance Angiogenesis and Osteogenesis of Human Adipose-Derived Stem Cells in Critical Tissue Defects. Biomacromolecules.

[18] Talaat WM, Haider M, Kawas SA, Kandil NG, Harding DR (2016). Chitosan-Based Thermosensitive Hydrogel for Controlled Drug Delivery to the Temporomandibular Joint. The Journal of craniofacial surgery.

[19] Upadhyaya L, Singh J, Agarwal V, Tewari RP (2014). The implications of recent advances in carboxymethyl chitosan based targeted drug delivery and tissue engineering applications. Journal of controlled release: official journal of the Controlled Release Society.

[20] Dragostin OM (2016). New antimicrobial chitosan derivatives for wound dressing applications. Carbohydrate polymers.

[21] H PSA (2016). A review on chitosan-cellulose blends and nanocellulose reinforced chitosan biocomposites: Properties and their applications. Carbohydrate polymers.

[22] Jayakumar R, Prabaharan M, Nair SV, Tamura H (2010). Novel chitin and chitosan nanofibers in biomedical applications. Biotechnology advances.

[23] Song L (2016). Peritoneal adhesion prevention with a biodegradable and injectable N,O-carboxymethyl chitosan-aldehyde hyaluronic acid hydrogel in a rat repeated-injury model. Scientific reports.

[24] Miranda DG (2016). A chitosan-hyaluronic acid hydrogel scaffold for periodontal tissue engineering. Journal of biomedical materials research. Part B, Applied biomaterials.

[25] Deng Y (2016). Evaluation of polypropylene mesh coated with biological hydrogels for temporary closure of open abdomen. Journal of biomaterials applications.

[26] Vertrees A (2008). Modern management of complex open abdominal wounds of war: a 5-year experience. Journal of the American College of Surgeons.

[27] Kaplan, M. Managing the open abdomen. *Ostomy/wound management* **50**, C2, 1–8, quiz 1p following 8 (2004). 14711999

[28] MacGillivray TE (2003). Fibrin sealants and glues. Journal of cardiac surgery.

[29] Christman KL, Fok HH, Sievers RE, Fang Q, Lee RJ (2004). Fibrin glue alone and skeletal myoblasts in a fibrin scaffold preserve cardiac function after myocardial infarction. Tissue engineering.

[30] Mohandas A, Anisha BS, Chennazhi KP, Jayakumar R (2015). Chitosan-hyaluronic acid/VEGF loaded fibrin nanoparticles composite sponges for enhancing angiogenesis in wounds. Colloids and surfaces. B, Biointerfaces.

[31] Zhu H (2006). The role of the hyaluronan receptor CD44 in mesenchymal stem cell migration in the extracellular matrix. Stem cells (Dayton, Ohio).

[32] Zhou B (2013). Rapidly in situ forming platelet-rich plasma gel enhances angiogenic responses and augments early wound healing after open abdomen. Gastroenterology research and practice.

[33] Takanari K (2017). Skeletal muscle derived stem cells microintegrated into a biodegradable elastomer for reconstruction of the abdominal wall. Biomaterials.

[34] Gurtner GC, Werner S, Barrandon Y, Longaker MT (2008). Wound repair and regeneration. Nature.

[35] Sell SA, McClure MJ, Garg K, Wolfe PS, Bowlin GL (2009). Electrospinning of collagen/biopolymers for regenerative medicine and cardiovascular tissue engineering. Advanced drug delivery reviews.

[36] Laschke MW (2006). Angiogenesis in tissue engineering: breathing life into constructed tissue substitutes. Tissue engineering.

[37] Bonafe F (2014). Hyaluronan and cardiac regeneration. Journal of biomedical science.

[38] Reeves AR, Spiller KL, Freytes DO, Vunjak-Novakovic G, Kaplan DL (2015). Controlled release of cytokines using silk-biomaterials for macrophage polarization. Biomaterials.

[39] Freytes DO, Kang JW, Marcos-Campos I, Vunjak-Novakovic G (2013). Macrophages modulate the viability and growth of human mesenchymal stem cells. Journal of cellular biochemistry.

[40] Vidal B (2008). Fibrinogen drives dystrophic muscle fibrosis via a TGFbeta/alternative macrophage activation pathway. Genes & development.

[41] Sica A, Mantovani A (2012). Macrophage plasticity and polarization: in vivo veritas. The Journal of clinical investigation.

[42] Mi Y, Su R, Fan DD, Zhu XL, Zhang WN (2013). Preparation of N,O-carboxymethyl chitosan coated alginate microcapsules and their application to Bifidobacterium longum BIOMA 5920. Materials science & engineering. C, Materials for biological applications.

[43] Chen SC (2004). A novel pH-sensitive hydrogel composed of N,O-carboxymethyl chitosan and alginate cross-linked by genipin for protein drug delivery. Journal of controlled release: official journal of the Controlled Release Society.

[44] Su WY, Chen YC, Lin FH (2010). Injectable oxidized hyaluronic acid/adipic acid dihydrazide hydrogel for nucleus pulposus regeneration. Acta biomaterialia.

[45] Ruhela D, Riviere K, Szoka FC (2006). Efficient synthesis of an aldehyde functionalized hyaluronic acid and its application in the preparation of hyaluronan-lipid conjugates. Bioconjugate chemistry.

[46] Yan S (2014). Injectable in situ self-cross-linking hydrogels based on poly(L-glutamic acid) and alginate for cartilage tissue engineering. Biomacromolecules.

