# Supplementary material for: Injectable in situ cross-linking chitosan-hyaluronic acid based hydrogels for abdominal tissue regeneration
Source: Sci Rep. 2017 Jun 2;7:2699. doi: 10.1038/s41598-017-02962-z (PMC5457437; doi:10.1038/s41598-017-02962-z)
Supplement: Supplementary file 1 — supplementary information [file 41598_2017_2962_MOESM1_ESM.pdf]

Supporting Information

Title

Injectable in situ cross-linking chitosan-hyaluronic acid based hydrogels for abdominal tissue regeneration

Youming Deng, Jianan Ren*, Guopu Chen, Guanwei Li, Xiuwen Wu, Gefei Wang, Guosheng Gu, Jieshou Li

Department of General Surgery, Jinling Hospital, Medical School of Nanjing University

Correspondence to: Jianan Ren, MD, FACS, Department of General Surgery, Jinling Hospital, Medical School of Nanjing University, 305 East Zhongshan Road, Nanjing 210002, Jiangsu Province, P. R. China. Tel: +86-25-80860437, Email: Jiananr@gmail.com

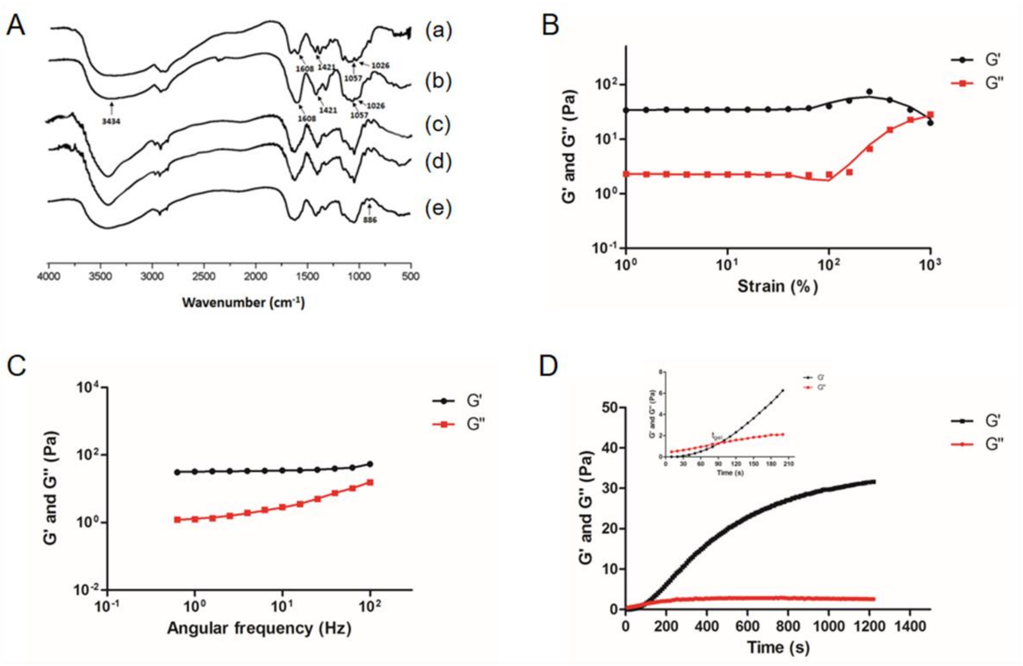

Fig S1 (A) FTIR spectra of CS (a), NOCC (b), HA (c), A-HA (d), and CS/HA hydrogel (e). Rheological characterization of 1/2 CS/HA hydrogels: (B) Strain-dependent (ω = 10 rad s−1) oscillatory shear rheology of the CS/HA hydrogel; (C) Frequency-dependent (at a strain of 1%) oscillatory shear rheology of the CS/HA hydrogel; (D) G’ and G” of the hydrogel measured over time at a strain of 1% and frequency of 10Hz. The gelation time (t_{gel}) was determined as the time point of crossover of G’ and G” curves.

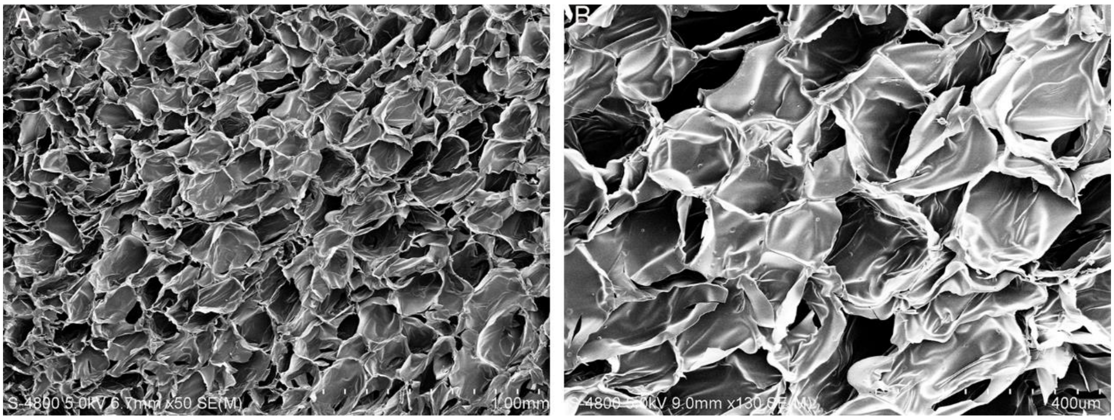

Fig S2 SEM images of 1/2 CS/HA hydrogel at 50× (A) and 130× (B) magnification.

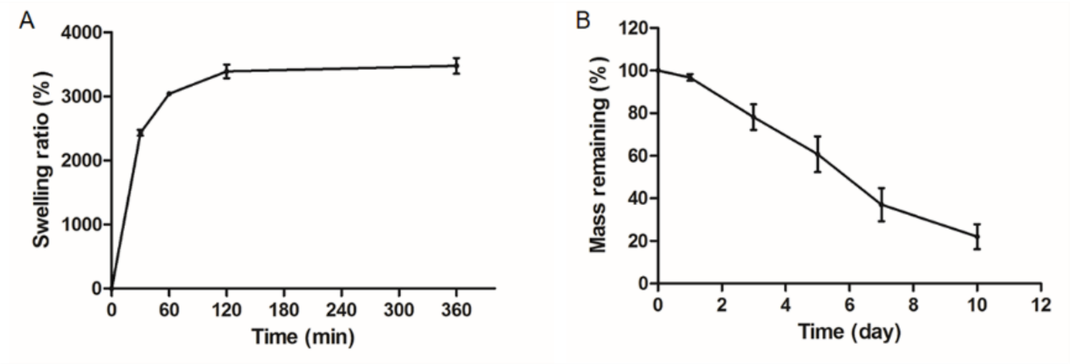

Fig. S3 Equilibrium swelling ratio of CS/HA hydrogel as a function of time incubated in PBS (pH= 7.4, 37℃). (B) In vitro degradation kinetics of the hydrogel in PBS (pH= 7.4, 37℃). Data were presented as mean ± SD (n= 3).

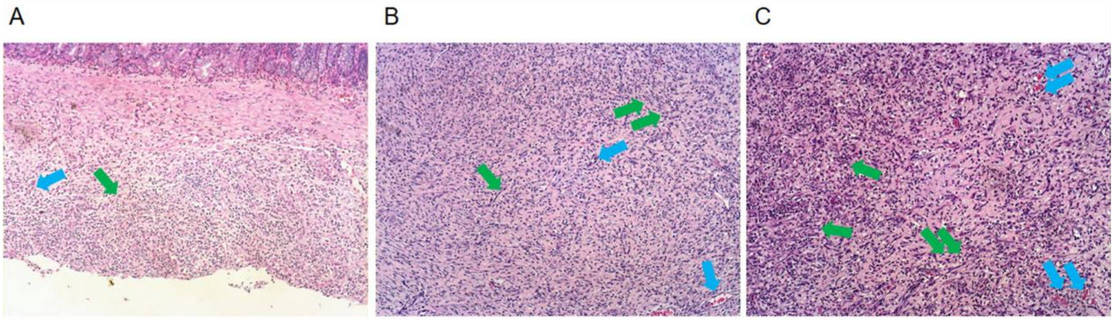

Fig. S4 Histologic appearance of the inner connective tissue in different groups: (A) the Control; (B) Fibrin gel; (C) CS/HA hydrogel. Blue arrows indicated vessels and green arrows indicated fibroblasts.

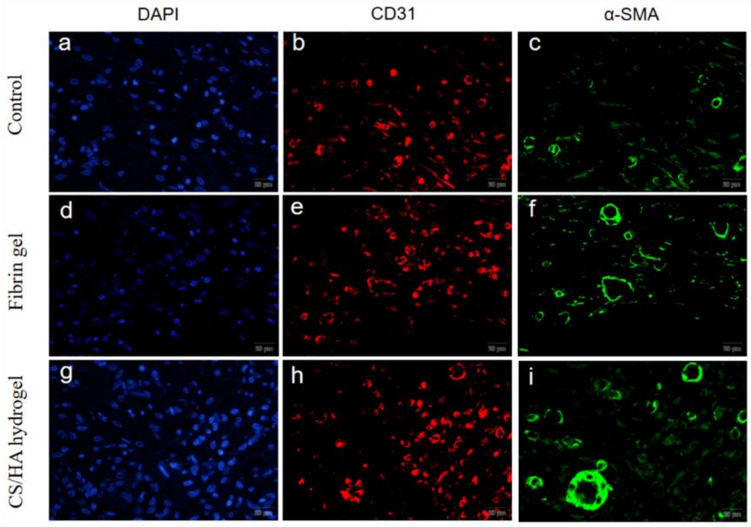

Fig. S5 Immunohistochemical staining for CD31 and α-SMA respectively in different groups. Dapi= blue, CD31= red, α-SMA= green.

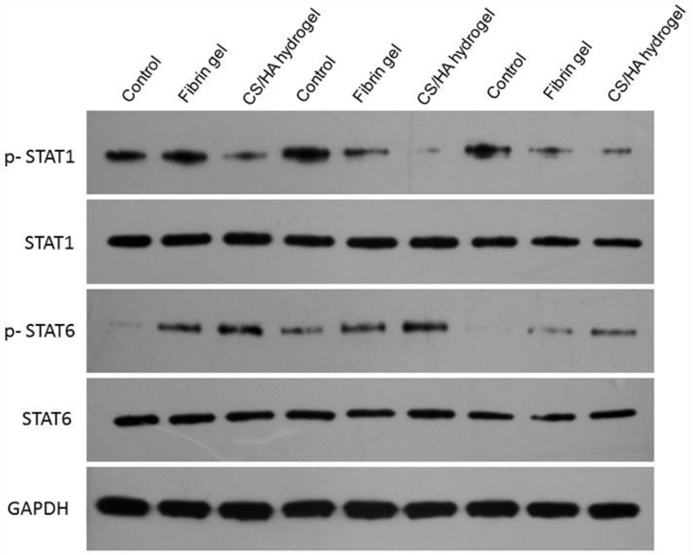

Fig. S6 full-length gels of expression of p-STAT1, STAT, p-STAT6 and STAT6 in Control, Fibrin gel and CS/HA hydrogel groups.

Table S1

Gelation time of different molar ratios of CS/HA hydrogel by test tube inverting method.

Molar ratio (NOCC/ A-HA)	Gelation time (s)
2:1	2100 ± 300
1:1	340 ± 60
1:2	90 ± 20

Table S2

Primer sequences of the genes involved in process of granulation tissue formation.

Genes	Forward	Reverse
IL-1	5’- CCTCGTCCTAAGTCACTCGC -3’	5’- GGCTGGTTCCACTAGGCTTT -3’
IL-4	5’- TCCACGGATGTAACGACAGC -3’	5’- TGGTGTTCCTTGTTGCCGTA -3’
IL-6	5’- CACTTCACAAGTCGGAGGCT -3’	5’- TCTGACAGTGCATCATCGCT -3’
TNF-α	5’- CATCCGTTCTCTACCCAGCC-3’	5’- AATTCTGAGCCCGGAGTTGG-3’
IL-10	5’- CGCTGTCATCGATTTCTCCC-3’	5’- TGCCGGGTGGTTCAATTTTT -3’
MCP-1	5’- TGATCCCAATGAGTCGGCTG-3’	5’- TGGACCCATTCCTTATTGGGG-3’
TGF-β	5’- CCATGACATGAACCGACCCT-3’	5’- TGCCGTACACAGCAGTTCTT-3’
VEGF	5’- GGGTCAAAAACGAAAGCGCA-3’	5’- TACACGTCTGCGGATCTTGG-3’
b-FGF	5’- CCTCGTCCTAAGTCACTCGC-3’	5’- GGCTGGTTCCACTAGGCTTT-3’
GAPDH	5’-GGCCTTCCGTGTTCCTACC-3’	5’- CGCCTGCTTCACCACCTTC-3’

IL: interleukin; TNF-α: tumor necrosis factor-α; VEGF: vascular endothelial growth factor; MCP-1: monocyte chemoattractant protein-1; TGF-β: transforming growth factor-β; bFGF: basic fibroblast growth factor.
